# Vegetable consumption and promotion among school-age children and adolescents in West Africa: a systematic review and narrative synthesis

**DOI:** 10.1017/S0007114524003301

**Published:** 2025-02-14

**Authors:** Kosisochukwu C. Igbokwe, Shirley I. Ejoh, Gideon O. Iheme

**Affiliations:** 1 Department of Human Nutrition and Dietetics, University of Ibadan, Ibadan, Nigeria; 2 Department of Food Studies, Nutrition and Dietetics, Uppsala University, Uppsala 753 10, Sweden; 3 Department of Human Nutrition and Dietetics, Michael Okpara University of Agriculture, Umudike, Nigeria

**Keywords:** Vegetable intake, Nutrition interventions, Malnutrition, Children, Micronutrient, Food systems

## Abstract

Low vegetable consumption among school-age children and adolescents puts them at risk of micronutrient malnutrition and non-communicable diseases. There is a dearth of synthesised literature on vegetable intake and interventions to promote increased consumption among this age group in West Africa. This study pooled evidence on vegetable consumption and interventions to promote vegetable consumption among school-age children and adolescents (6–19 years) in West Africa. Quantitative and qualitative studies from 2002 to 2023 were electronically searched in PubMed, African Journals Online (AJOL) and Google Scholar databases. The Preferred Reporting Items for Systematic Reviews and Meta-Analyses system was adhered to in reporting this review **(**PROSPERO ID: CRD42023444444). The Joanna Briggs Institute critical evaluation tool was used to appraise the quality of studies. Forty (40) studies met the search criteria out of *n* 5080 non-duplicated records. Meta-analysis was not possible due to high heterogeneity. Low vegetable consumption expressed in frequency or amounts was recorded among school-age children and adolescents in the reviewed studies. Intervention studies were mostly among adolescents; the most common type of intervention was the use of nutrition education. Insufficient evidence and high heterogeneity of studies reflect the need for more high-quality interventions using globally identified standards but applied contextually. School-age children appear to be an under-served population in West Africa with regard to nutrition interventions to promote vegetable consumption. There is a need for multi-component intervention studies that encourage vegetable consumption as a food group. Gardening, parental involvement, gamification and goal setting are promising components that could improve the availability, accessibility and consumption of vegetables.

School age represents an important developmental stage of life and the second window of opportunity to consolidate the health and nutrition gains made after early childhood for growth, psychosocial development and establishing lifelong dietary and lifestyle habits and preparation for pubertal life (adolescence)^(1)^. Good nutrition during this phase is essential to ensuring optimal growth and development as well as improving short and long-term health outcomes. Children’s nutrition is particularly important, not only because many eating habits that are formed in childhood will persist into adulthood but also because nutrition plays a role in preventing chronic diseases^(2)^. In order to maximise long-term health outcomes, it is also crucial to support children in developing good eating habits, as this is pivotal to support the adoption of healthy eating behaviours at this stage^(3)^. There is an increasing awareness that children’s eating behaviours are influenced by environmental factors: home environment and parental influence, as well as the school environment, are recognised as major contributors to the eating habits of children^(4,5)^.

Due to the dynamic nature of their growth and development, school-age children (SAC) and adolescents have an increased need for nutrients^(6)^. This should include nutrients needed to support physical and cognitive growth and development, offer sufficient energy reserves for illnesses and pregnancy and avoid the adult onset of nutrition-related diseases^(7)^.

Several population groups particularly SAC and adolescents appear to be excluded in most global and regional data related to nutrition, with only under-5 children, women of reproductive and adults prominently captured^(8)^. This gap results in limited insights into the food consumption habits of these overlooked age groups, despite the existence of various national interventions aimed at addressing their nutritional needs^(9)^.

Low fruit and vegetable (FV) consumption increases the risk of micronutrient deficiencies and non-communicable illnesses, which are known to be major causes of death globally^(10,11)^. For example, childhood overweight and obesity are associated with low consumption of certain nutrient-rich foods and excessive consumption of nutrient-poor, high-calorie foods^(12)^. The number of children and adolescents who are overweight or obese has more than doubled over the past 50 years^(13,14)^. Thus, SAC and adolescents’ diets low in FV may deprive them of micronutrients essential for growth, development and bodily functions, which increase their risk of developing non-communicable diseases later in life^(15)^. Hence promoting increased consumption of FV among SAC and adolescents is of public health importance.

Western Africa is one of the youngest populations in the world with more than a tenth of its population estimated to be below 15 years of age^(16)^. In this region like other low- and middle-income countries, many SAC do not meet dietary recommendations for FV^(17–20)^. Sub-Saharan Africa has been found to be the region with one of the highest levels of micronutrient deficiencies in under-5 children and women comparable only to South Asia^(21)^. Although intake levels of FV for all age groups in Africa except North Africa are well below the recommended standards^(22)^, Western Sub-Saharan Africa has the highest age-standardised prevalence rates of dietary iron deficiency than other regions in Africa, which fruits and vegetables are the primary plant-based sources^(21)^. These global and regional evidence are also supported by studies within different locations in the Western African region where insufficient amounts of fruits and vegetables were reported^(17,23,24)^. It is probable that most of the poor/low intake may be more for vegetables, as studies have shown that children’s preferences for vegetables in particular are consistently lower than for fruits^(25)^.

The period of school age and adolescence are critical stages where attitudes, knowledge and skills acquired can influence their behaviour in adulthood^(26)^. This then offers a window of opportunity for interventions to build their capacity to acquire healthy eating habits and improve their vegetable intake, to prevent micronutrient malnutrition and the onset of diet-related chronic diseases in later life, associated with poor and unhealthy dietary patterns and practices earlier in life. Hence there is a need to take a critical look at the vegetable intake of the target population and also intervention studies to promote their consumption. However, when compared with other lifecycle stages, limited research and intervention studies have focused on the health and nutrition of SAC^(27)^. For adolescents, there is a lot of focus on their reproductive health. There are very few interventions to address the poor intake of vegetables and fruits among SAC and adolescents. To the best of the authors’ knowledge, a comprehensive review of the literature specifically studying vegetable intake and interventions to promote health-related behaviour to improve vegetable intake among SAC and adolescents to support these assumptions in the West Africa sub-region, where regional evidence suggests priority attention is needed, has not been undertaken.

Therefore, the purpose of this study was to perform a comprehensive systematic review and provide an up-to-date summary to answer the following questions: (1) What is the vegetable intake and consumption pattern of SAC and adolescents in West Africa, including the methodologies/assessment tools employed? (2) What are the common interventions that have been used to promote health-related behaviour to improve vegetable intake among SAC and adolescents in West Africa? (3) What are the barriers and facilitators to vegetable consumption and promotion among SAC and adolescents in West Africa? It is hoped that the results from this review may be used to guide future research and inform intervention studies for promoting increased vegetable consumption among SAC and adolescents.

## Methods

A systematic review of the literature of qualitative and quantitative studies was conducted according to a pre-specified protocol that was registered with the International Prospective Register of Systematic Reviews (PROSPERO record with the ID CRD42023444444). The Preferred Reporting Items for Systematic Reviews and Meta-Analyses system was adhered to in the reporting of this review.

### Search strategy

The search took place between 16 December 2022 and 28 March 2023. For primary research publications published between the years 2002 and 2022, three databases were used: PubMed, African Journals Online (AJOL) and Google Scholar. We did not conduct a search of the grey literature since we preferred to include only works that had been peer-reviewed, published and available online. The following keyword combinations were used in each of the various databases advanced search features: (Vegetable OR micronutrient) AND (Intake OR consumption OR diet OR dietary OR eating OR nutrition) AND (Primary school children OR Adolescent OR school-age children OR secondary school student OR teen OR pupil) AND (Nigeria OR Benin OR Burkina Faso OR Cabo Verde OR Cote d’Ivoire (Ivory Coast) OR Gambia OR Ghana OR Guinea OR Guinea-Bissau OR Liberia OR Mali OR Mauritania OR Niger OR Senegal OR Sierra Leone OR Togo).

### Inclusion and exclusion criteria

Studies were considered eligible for inclusion if they met the following criteria:

Population: SAC and adolescents in the West African region between the ages of 6 and 19 years were included. Studies published in English were included.

Outcomes: Studies were included if they reported vegetable consumption, promotion of vegetables or the role of micronutrients from vegetables.

Study design: Experimental (intervention studies) and observational (cross-sectional, cohort and case control) were eligible.

Systematic reviews, unpublished theses, case studies, conference abstracts, special population studies (participants characterised with an illness or a problem), population studies involving children under 6 and those involving adults aged 19 and older and studies with low risk of bias or written in another language other than English were all excluded. Studies whose conclusions did not take into account vegetable intake, promotion of vegetables or the role of micronutrients were excluded.

### Study selection

The article titles were screened to find publications that were pertinent. The paper’s alignment with the necessary inclusion and exclusion criteria was then verified by reading the abstract of pertinent articles by two of the researchers. The articles whose abstracts either satisfied these requirements or fell short of effectively describing the specifics were then downloaded and reviewed in their entirety.

### Data collection/extraction

Utilising tables, the data were individually retrieved and recorded using Microsoft Excel by two of the researchers. Missing information was noted as unavailable. The following information was included in the data that were extracted: title, author(s), country, city or location, sample population, methodology (sample size, study design, sampling techniques, variables and study instruments), data analysis, important findings and conclusion. All discrepancies were discussed and resolved by all three researchers.

### Quality assessment/appraisal

The risk of bias assessment for the included studies was independently conducted by two of the researchers. The Joanna Briggs Institute critical evaluation tools that were appropriate for this review’s eligible study designs were applied to each included document to help determine whether to include, exclude or request more information on a particular study^(28)^. The Joanna Briggs Institute checklist, which consists of eight questions, was used to assess the methodological quality of research by identifying the number of potential biases in the design, conduct, analysis and write-up based on the range of our eligible designs. The eight questions focus on *(1) study based on random/pseudo-random sample, (2) clear definition of study inclusion criteria, (3) description of study subjects and settings, (4) outcomes/exposures measured in a valid and reliable way, (5) identification of co-founding factors and strategies, (6) sufficient descriptions for comparison groups, (7) follow-up done for a sufficient time period and (8) appropriate statistical analysis* ^(28)^. There are four possible responses to each question: ‘yes’, ‘no’, ‘unclear’ and ‘NA’ (non-applicable). Owing to the varying design of the studies reviewed, the researchers focused on identifying criteria common across all of them. These studies were rated positive for inclusion when at least 50 % of each of the five common criteria – 1, 2, 3, 4 and 8 – were met. However, fulfilling all the criteria would result in a total score of 8 (1 point per criterion). The risk of bias was categorised into three levels: low quality (0–2), medium quality (3–5) and high quality (6–8).

Similar to the screening, the methodological quality of the eligible studies was evaluated, and all disagreements were resolved by consensus of the researchers or consultation of a third reviewer.

### Data analysis and synthesis

Data were deductively grouped and analysed according to the relevant research questions: (i) vegetable intake and consumption pattern of SAC and adolescents in West Africa (outcome variables, measurement tools, study outcome/frequency of vegetable consumption), (ii) interventions that have been used to promote vegetable intake among SAC and adolescents and (iii) barriers and facilitators of vegetable consumption.

## Results

**Figure 1.**
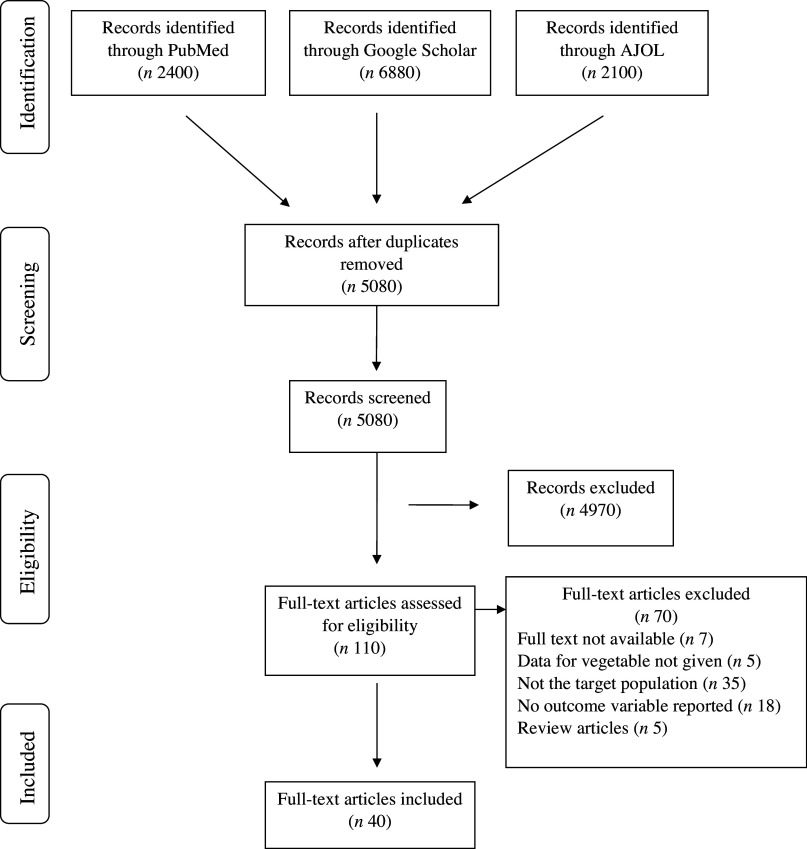
Flow chart of article selection based on Preferred Reporting Items for Systematic Reviews and Meta-Analyses.

A total of 11 380 possibly relevant articles were found in three (3) databases after the literature search (Fig. 1). 5080 articles remained after duplicate records were removed. 4970 records were eliminated after those articles’ eligibility was checked for the title and abstract. The remaining 110 documents were evaluated and scrutinised in their entirety. Full-text screening resulted in the exclusion of seventy articles. Thus, forty articles in all were finally included in this review.

### Description of study characteristics

In total, 24 391 SAC and adolescents recruited mostly from schools in five different countries were included in this review. Most of the studies were mainly reported from the population of Nigeria (*n* 26). Sample size ranged from 18 to 2786 participants with more than 75 % (30 out of 40) of the included studies recruiting between 101 and 1000 participants. The outcomes of each study varied, but the majority primarily focused on the frequency of vegetable consumption. An overview of the included studies is presented in Table 1, while the summary of study characteristics is presented in Table 2.

**Table 1.** Overview of the included studies

Authors, year and location	Sample size	Region/area	Study population	Study design	Outcome variables	Measurement tools	Study outcome (frequency of vegetable consumption)
Ilo *et al.*, 2022 Nigeria^(29)^	227	Rural	School-age children (6–12 years)	Cross-sectional	Food frequency pattern of the children	FFQ	Green leafy vegetable (GLV) consumption – 61·0 %
Anyiam *et al.*, 2022 Nigeria^(30)^	384	Urban	School-age children (6–12 years)	Community-based cross-sectional	Food intake/frequency of consumption from each food group	FFQ (on selected food groups) and 24 h dietary recall	Vegetable consumption is consumed Occasionally – 0·52 % < 3×/week – 25·52 % 4–6×/week – 44·5 % Daily – 29·4 %
Amu *et al.*, 2017 Nigeria^(31)^	206	Urban	School-age children (8–10-year-old)	Community-based comparative cross-sectional	24 h dietary pattern	24 h dietary recall	Public school: 2×/week – 24·3 % Once/week – 28·2 % Occasionally – 47·6 % Private school: 2×/week – 21·3 % Once/week – 23·3 % Occasionally – 55·3 %
Fadeiye and Adekanmbi, 2020 Nigeria^(32)^	260	Sub-urban	School-age children and adolescents (6–16-year-old)	Cross-sectional	Fruits and vegetables consumed and their rate of consumption on a weekly basis	FFQ	Result showed that tomatoes (96·5 %), onions (94·2 %), carrot (92·7 %), okra (88·1 %) and bitter leaf (80·4 %) were mostly consumed.
Adeomi *et al.*, 2020 Nigeria^(33)^	260	Urban	School-age children and adolescents (5–19-year-old)	Cross-sectional study	Frequency of food consumption	24 h dietary recall	In the week preceding the week of study, vegetable consumption was ≤ 3×/week – 20·0 % > 3×/week – 80·0 %
Agugo *et al.*, 2019 Nigeria^(34)^	115	Urban	School-age children (5–13-year-old)	Cross-sectional	Feeding patterns in each home from the caregivers	54-item FFQ	2–3×/week – 71·4 %
John-Akinola *et al.*, 2021 Nigeria^(35)^	728	Urban	School-age children and adolescents (6–15-year-old)	Cross-sectional	Food contents in lunch boxes were observed using a checklist.	24 h dietary recall	21 %
Adeniyi *et al.*, 2019 Nigeria^(36)^	440	Urban	School-age children (6–13-year-old)	Community-based cross-sectional	Food consumption pattern	Not stated	0·04 %
Akinola *et al.*, 2022 Nigeria^(37)^	1120	Urban	Adolescents (10–19-year-old)	Cross-sectional	Dietary habits	4-item FFQ	Never – 11·8 % 1 portion/d – 33·2 % 2–3 portions/d – 55·0 %
Olumakaiye, 2013 Nigeria^(38)^	600	Urban	School-age children (6–11-year-old)	Cross-sectional	Dietary diversity measures	24 h dietary recall and dietary diversity questionnaire composed of sixteen food groups	Private school: Vitamin A-rich veggie/tuber – 32·3 % Dark GLV – 50·3 % Other vegetables – 100·0 % Public school: Vitamin A-rich veggie/tuber – 7·7 % Dark GLV – 48·7 % Other vegetables – 100·0 %
Ayogu, 2019 Nigeria^(39)^	90	Rural	School-age children and adolescents (6–15-year-old)	Cross-sectional	Dietary diversity, nutrient intake	24 h dietary recall and dietary diversity questionnaire	32·1 %
Ibeanu *et al.*, 2020 Nigeria^(40)^	869	Sub-urban	Adolescents (13–17-year-old)	Quasi-experimental study design with one intervention group	Consumption pattern of micronutrient-rich foods		The study revealed a percentage increase in the proportion of respondents who consumed carrots (336·34 %) and leafy vegetables (85·56 %) daily after the intervention.
Menakaya and Menakaya, 2022 Nigeria^(41)^	18	Urban	Adolescents (18–19-year-old)	Qualitative study	Dietary pattern	Interview guide/in-depth interview	Study participants described their attitudes and practices towards a healthy lifestyle in terms of personal conviction and disposition, regular physical activity and consumption of fruits and vegetables.
Silva *et al.*, 2017 Nigeria^(42)^	220	Urban	Adolescents (10–16-year-old)	Cross-sectional	Consumption, facilitators and barriers to consumption of fruits and vegetables	Not indicated	Consumption of fruits and vegetables was appropriate in only 5·48 % of the respondents, having five portions of fruits and vegetables daily.
Anaemene and Ogunkunle, 2020 Nigeria^(43)^	478	Urban	School-age children (8–11-year-old)	Cross-sectional	Dietary habits	24 h dietary recall	School 1 – 86·2 % School 2 – 72·1 % School 3 – 72·9 % School 4 – 64·6 %
Olatona *et al.*, 2020 Nigeria^(44)^	682	Urban	Adolescents (10–19 year)	Cross-sectional	Dietary habits, nutrient intake	15-item FFQ and 24 h dietary recall	11·4 % consumed vegetables, while only 9·7 % of the adolescents consumed adequate fruits and vegetables (400 g or 5 servings) daily.
Shapu *et al.*, 2022 Nigeria^(45)^	417	Urban	Adolescents (10–19-year-old) – female only	Cluster randomised control trial	Dietary practice of respondents	12-item FFQ	There was a statistically significant difference at 3 and 6 months post-intervention for dietary practice; *P* = 0·003 and *P* = 0·011 between the intervention and control groups.
Ogunkunle and Oludele, 2013 Nigeria^(46)^	302	Semi-urban	Adolescents (10–19-year-old)	Cross-sectional	Food intake and meal patterns	FFQ (seven food groups) and 24 h dietary recall	Daily – 26·4 % 4–6×/week – 2·8 % < 3×/week – 27·5 % Occasionally – 43·3 %
Wordu and Wachukwu- Chikodi, 2019 Nigeria^(47)^	150	Urban	Adolescents (10–19-year-old)	Cross-sectional	Eating patterns, lifestyle characteristics	Not indicated	Fruits and vegetables: Once/week – 16·7 % 2–3×/week – 16·7 % 4–5×/week – 13·3 % Daily – 23·3 % More/week – 30·0 %
Uba *et al.*, 2020 Nigeria^(48)^	250	Rural	Adolescents (13–19-year-old) – female only	Cross-sectional	Dietary habits	Not indicated	≤ 2×/week – 44·8 % > 2×/week – 55·2 %
Wordu and Orisa, 2021 Nigeria^(49)^	236	Urban	Adolescents (10–16-year-old)	Cross-sectional	Food consumption pattern	11-item FFQ	Never – 2·97 % 1–2×/week – 38·14 % 3–4×/week – 13·14 % > 5×/week – 16·10 % Daily – 29·66 %
Nnebue *et al.*, 2016 Nigeria^(50)^	300	Urban	School-age children (5–12-year-old)	Cross-sectional	Feeding practices of the children	24 h dietary recall	The 24 h nutritional dietary intake recall showed that 279 (93·0 %) took more of carbohydrates. Only 179 (59·0 %) had farms in their homes.
Sanusi *et al.*, 2015 Nigeria^(51)^	393	Urban	Adolescents (10–19-year-old)	Cross-sectional	Food and food groups consumed	24 h dietary recall and dietary diversity questionnaire	Total (fruits and GLV) – 67·4 % males – 64·4 % Females – 69·9 %
Ezerika *et al.*, 2018 Nigeria^(52)^	31	Urban	Adolescents (13–17-year-old)	Qualitative study	Knowledge about nutrition		Participants reported that the intervention shifted their perception and preferences, leading them to alter their behaviour by incorporating more nutritious foods (such as fruits and vegetables) into their diet.
Seidu *et al.*, 2021 Ghana^(53)^	2786	Urban	Adolescents (10–19-year-old)	Cross-sectional analysis of data from Global School-based Student Health Survey	Fruit consumption, vegetable consumption and adequate fruit and vegetable consumption	FFQ	26·8 %
Yaméogo *et al.*, 2018 Burkina Faso^(54)^	1993	Urban	Adolescents (10–19-year-old)	Cross-sectional	Vegetable intake	24 h dietary recall and FFQ	10·0 %
Giguère-Johnson *et al.*, 2021 Senegal^(55)^	13	Urban	Adolescents (14–16-year-old) – female only	Cross-sectional	Food intake and eating behaviours	24 h dietary recall (fourteen food groups)	4 % (WHO recommendations of 25 g for fibre was used)
Nago *et al.*, 2010 Benin^(56)^	656	Urban	Adolescents (13–19-year-old)	Cross-sectional	Food composition pattern	24 h dietary recall (ten food groups)	Total (fruits and vegetables) – 6 % Daily – 26 % (out-of-home foods) Daily – 74 % (in-home prepared foods)
Owusu *et al.*, 2007 Ghana^(57)^	140	Selected schools from urban and rural areas	Adolescents in boarding school (14–18-year-old)	Cross-sectional	Eating pattern	81-item FFQ	2 servings of vegetables/week
Sagbo *et al.*, 2022 Benin^(58)^	612	Rural and urban area of Lokossa	School-age children and adolescents (8–17-year-old)	Cross-sectional	Family and school context and frequency of healthy and unhealthy food consumption	FFQ	> 5×/week – 32·0 % < 5×/week – 68·0 %
Doku *et al.*, 2011 Ghana^(59)^	1566	Rural and urban	Adolescents (12–18-year-old)	Cross-sectional	Food habits	FFQ	Never – 13·0 % 1–3×/week – 34·0 % 4–6×/week – 14·0 % Daily – 38·0 %
Abizari *et al.*, 2017 Ghana^(60)^	228	Rural area of Tolon district	School-age children (6–12-year-old)	Cross-sectional study conducted in different seasons – dry season (Oct 2010) and rainy season (May 2011)	Dietary diversity score	24 h dietary recall based on 13 food groups	Dry season: Vitamin A-rich dark GLV – 23·3 % Vitamin A-rich deep orange, yellow and red vegetable – 73·7 % Vitamin C-rich vegetable – 96·5 % All other fruits and vegetables – 93·4 % Rainy season: Vitamin A-rich dark GLV – 52·6 % Vitamin A-rich deep orange, yellow and red vegetable – 36·4 % Vitamin C-rich vegetable – 100·0 % All other fruits and vegetables – 90·8 %
Hormenu, 2022 Ghana^(61)^	1311	Rural	Adolescents (10–15-year-old)	Cross-sectional	Dietary practices	6-item FFQ	Fruits and vegetables – 49·9 %
Dabone *et al.*, 2013 Burkina Faso^(62)^	769	Urban and Peri-urban	School-age children (6–12-year-old)	Cross-sectional	Consumption frequency of ‘healthy’ foods	FFQ	Never – 17·0 % 1–2×/week – 42·9 % 3–4×/week – 27·7 % 5–6×/week – 8·3 % Daily – 4·0 %
Fiorentino *et al.*, 2016 Senegal ^(63)^	545	Urban	School-age children and adolescents (5–17-year-old)	Cross-sectional	Dietary intake	24 h dietary recall	For all micronutrients, at least half of the children had insufficient intake, suggesting a diet poor in fruit and vegetables, with a special concern for Zn, vitamin A, folic acid and Ca.
Alangea *et al.*, 2018 Ghana ^(64)^	487	Urban	School-age children and adolescents (9–15-year-old)	Cross-sectional	Dietary behaviour and food consumption patterns	100-item FFQ	Starchy root with vegetable dietary pattern was negatively associated with overweight/obese status, private school attendance and higher socio-economic status (SES) after controlling for age at the bivariate level.
Nago and Chabi, 2019 Benin ^(65)^	229	Urban	Adolescents (13–19-year-old)	Pilot intervention using a pre-post without control	Dietary intake	24 h dietary recall	The contribution of fruit consumption at school to consumers’ daily fruit intake rose from 3 % at baseline to 78 %.
Otuneye *et al.*, 2017 Nigeria^(66)^	1550	Urban and Rural	Adolescents (10–19-year-old)	Cross-sectional	Dietary habits, knowledge of nutrition	FFQ	Urban school setting – 70·3 % Rural school setting – 64·9 %
Uzosike *et al.*, 2020 Nigeria^(67)^	847	Urban	School-age children (6–11-year-old)	Cross-sectional	Dietary diversity and dietary pattern	24 h dietary recall	Dark GLV – 41·0 % Vitamin A-rich fruits and vegetables – 17·1 % Other fruits and vegetables – 7·7 %
Schreinemachers *et al.*, 2019 Burkina Faso ^(68)^	1760	Rural	School-age children and adolescents (8–14-year-old)	Repeated cluster randomised controlled trial	Fruit and vegetable preference	24 h dietary recall	There was no significant increase in other outcome indicators including fruit and vegetable consumption.

**Table 2.** Summary of characteristics of studies

Characteristics	Total		References
	*n*	%	
Publication year			
2002–2012	3	7·5 %	^(56,57,59)^
2013–2022	37	92·5 %	^(29–55,58,60–68)^
Country			
Benin	3	7·5 %	^(56,58,65)^
Burkina Faso	3	7·5 %	^(54,62,68)^
Ghana	6	15·0 %	^(53,57,59–61,64)^
Nigeria	26	65·0 %	^(29–52,66,67)^
Senegal	2	5·0 %	^(55,63)^
Number of participants			
Less than 100	3	7·5 %	^(39,41,52)^
101–1000	30	75·0 %	^(29–36,38,40,42–51,55–58,60–65,67)^
1001 and above	7	17·5 %	^(37,53,54,59,61,66,68)^
Sex			
Studies with males and females	37	92·5 %	^(29–44,46,47,49–54,56–68)^
Females – only study	3	7·5 %	^(45,48,55)^
Assessment tools			
FFQ	14	35·0 %	^(29,32,34,37,45,49,53,57–59,61,62,64,66)^
24 h dietary recall	12	30·0 %	^(31,33,35,43,50,55,56,60,63,65,67,68)^
FFQ + 24 h dietary recall	4	10·0 %	^(30,44,46,54)^
In-depth interview	1	2·5 %	^(41)^
24 h dietary recall + dietary diversity	3	7·5 %	^(38,39,51)^
Not stated	6	15·0 %	^(36,47,48,52,40,42)^

### Quality assessment of included studies

**Figure 2.**
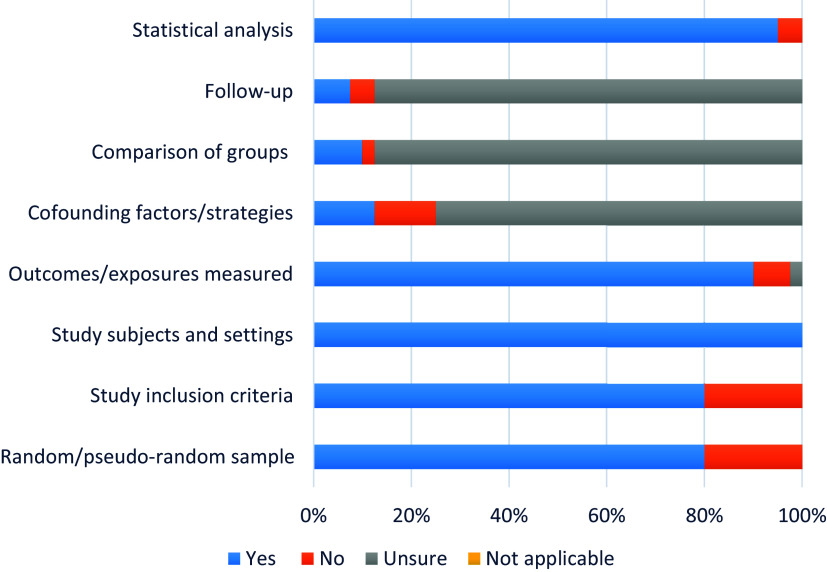
Quality assessment of included studies using the Joanna Briggs Institute checklist.

The quality assessment/appraisal using Joanna Briggs Institute critical appraisal checklist for descriptive/case studies as presented in Fig. 2 showed that 80 % of the reviewed studies were based on random/pseudo-random sample and had clear definition of study inclusion criteria, respectively. All studies had a clear description of the study subjects and setting, 90 % had their outcomes measured in a valid and reliable way, while 12·5 % identified other confounding factors and strategies. For the reviewed qualitative studies, 10 % gave sufficient description for comparison of groups, while follow-up done for a sufficient time period was 7·5 %. Almost all (95 %) of the studies were analysed appropriately. In general, six (15 %) were assessed as high-quality, thirty-four records (85 %) as medium quality and none for low quality, indicative of reliable evidence and low risk of bias. Detailed quality appraisal of each study is reported in the online Supplementary material Table 1.

### Dietary assessment methodologies

Assessment tools in the included studies primarily consisted of FFQ (*n* 14). The number of items in the FFQ included 4-item FFQ^(37)^, 54-item FFQ^(34,43)^, 12-item FFQ^(45)^, 11-item FFQ^(49)^, 81-item FFQ^(57)^, 6-item FFQ^(61)^ and 100-item FFQ^(64)^. In addition, a 24 h dietary recall was used in twelve of the studies. In six studies, FV consumption data were merged together^(42,47,48,56,61,66)^. Three of the studies used the Global School-based Student Health Survey questionnaire^(58,61,62)^, and eight used validated and acceptable questionnaires/tools for national surveys^(38,41,45,48,51,55,56,67)^. One of these studies^(45)^ used an online questionnaire (KoBo Collect) to collect data. Questionnaire piloting was reported in nineteen of the studies. In comparison, six studies did not report the type of dietary assessment measured (quantity, frequency, etc.). The WHO-recommended five servings of fruits and vegetables were used as the adequacy cutoff point in two of the studies^(42,43)^ reporting the percentage of SAC and adolescents meeting the recommendation. Nonetheless, other cutoff points were used in a few studies. One of the studies^(54)^ defined inadequacy as intake of < 1 slice of fruit or < 1 portion of raw or cooked vegetable per d. Another study^(36)^ established their cutoff mark as two servings of FV, while another^(55)^ used the WHO recommendation of 25 g of fibre.

### Dietary outcome (vegetable consumption pattern)

Vegetable consumption data were presented in means, percentage of students consuming regularly or irregularly and those meeting the recommendations and frequencies. Mean consumption was recorded among four countries. A study by Giguère-Johnson *et al.* ^(55)^ conducted in Senegal reported mean vegetable consumption as 32 ± 44 g/d, while a study conducted in Benin^(56)^ reported a mean consumption of 97 g/d. In Burkina Faso^(62)^, vegetable consumption was reported to be 2·3 times/week and 1·68 servings/week as observed by Owusu *et al.* ^(57)^ in Ghana.

### Interventions that promote vegetable intake

Five of the reviewed studies^(40,45,52,65,68)^ evaluated interventions that promote vegetable intake among SAC and adolescents as shown in Table 3. These include school garden and complementary education, nutrition education, gamification, school-based fruit stall and health education. Two of the reviewed studies^(29,50)^ found an increase in vegetable consumption and dietary habits among the respondents due to their parents owning a home garden.

**Table 3.** Interventions that promote vegetable intake among school-age children and adolescents

Study and country	Study population/sample size	Study design	Duration of the study	Intervention	Components of the intervention	Outcomes of the study
Ibeanu *et al.*, 2020 Nigeria^(40)^	Adolescents/869 (pre-test) and 776 (post-test)	Quasi-experimental study with one intervention group	3 weeks intervention and 6 months post-intervention	Nutrition education	Nutrition education aids developed with nutrition facts, pictures of micronutrients-rich foods and computer graphics compiled into a fourteen-page booklet and include definitions of food nutrients, classes and amount required, food sources of micronutrients of interest, functions of the micronutrients, signs and symptoms of the micronutrient deficiencies and inhibitors of the micronutrient absorption.	Increase in nutrition knowledge and consumption of some micronutrients-rich food sources including leafy and non-leafy (carrot) vegetables among adolescents
Shapu *et al.*, 2022 Nigeria^(45)^	Adolescents/403	Cluster randomised control trial	3 months intervention and post-intervention at 3 and 6 months	Health education	How to prevent malnutrition through information on macronutrients, micronutrients, dietary diversity and healthy eating, motivation on the prevention of malnutrition among adolescent girls and lessons learnt and behavioural skills on practical cooking demonstration and identification of food groups.	The health education intervention greatly impacted dietary practice among adolescent girls in Maiduguri Metropolitan Council.
Nago and Chabi, 2019 Benin^(65)^	Adolescents/249	Pre-post design without control	2 months	School-based fruit stall	A single leaflet about the general health benefits of fruits and vegetables was distributed to the students and teachers and sent home to the parents.	The intervention is promising and could be an efficient and sustainable means to promote fruit consumption and healthy diet in adolescents in urban Benin.
Schreinemachers *et al.*, 2019 Burkina Faso^(68)^	School-age children and adolescents/1760	Repeated cluster randomised controlled trial	6 months	School gardens and complementary nutrition education	A school garden for the cultivation of locally accepted vegetables. Seed of a range of vegetables was provided together with gardening tools and other equipment as needed. The second component involved complementary education about agriculture, nutrition and water, sanitation and hygiene. Topics covered were food groups, the health benefits of vegetables, food and body hygiene and school and environmental sanitation. The third component was the involvement of parents, local farmers and other community members in the school garden.	Significant increases in children’s knowledge about sustainable agriculture and about food and nutrition but did not lead to significant improvements in children’s fruit and vegetable intake
Ezerika *et al.*, 2018 Nigeria^(52)^	Adolescents/31	Semi-structured Focus group discussion	4–12 weeks	Gamification	The core strategy of the game is to buy healthy food cards in order to get as many points as possible. Points gained in the game translate into vouchers through a voucher system integrated into the game that enables players to buy real food (fruits and non-leafy vegetables) from partnering tuck shops.	The results from the focus groups suggest that gamification of nutrition can lead to improvements in dietary behaviour among adolescents over the short term.

### Barriers/facilitators of vegetable consumption

Three of the reviewed studies implemented in a school environment assessed the barriers/facilitators influencing vegetable consumption, while one study looked at the behavioural determinants of healthy food consumption (FV). Three studies reported on facilitators only, and two of them were studies from Nigeria^(32,41)^ and the third one from Burkina Faso^(62)^. One study from Nigeria^(42)^ reported both facilitators and barriers. The general observation from the reviewed studies was that vegetable consumption was facilitated by availability and accessibility at home and in school; consumption by parents, siblings and peers; and its health benefits. Specific comments captured were ‘*it being served at home, siblings like vegetable, like the taste of vegetables and like for home-made stew*’.

‘*her mum loves taking fruits and she joins her; that’s how she developed the habit of taking fruits and vegetables*’. Barriers to vegetable consumption reported were preparation time lack of taste and attractiveness as well as not making one feel full after consuming it.

## Discussion

In this review, studies on vegetable promotion and consumption among SAC and adolescents in West Africa were comprehensively examined. The review included forty studies in total, and these publications included information on the frequency of vegetables consumed by country as well as interventions that support increasing vegetable consumption among different age groups. Due to the high heterogeneity of the reported studies consulted, a meta-analysis was not possible. The results of this systematic review reveal that most studies lumped the intake of fruits and vegetables together, and within the vegetable group, the types were not disaggregated either. This has implications for providing accurate results on the adequacy or lack thereof of vegetable consumption among the age group studied.

### Vegetable intake/consumption pattern of school-age children and adolescents

The observed disparities in data assessment tools and techniques for vegetable consumption in different studies have been previously mentioned by several authors and expert groups^(69–71)^. These reports posited that the unavailability of harmonised dietary assessment indicators poses a critical gap in the comparability of findings and pooling of evidence to compensate for this missing global evidence for the target population in question. The varied FFQ items ranging from 4 to 100 further strengthen the case for more standardisation/harmonisation of FV dietary indicators. However, this is more challenging as other studies have affirmed that the reproducibility of FFQ varies between the FFQ items and age groups, and lower reproducibility is often found among children and adolescents than among adults and the elderly^(72)^.

The low consumption of vegetables (at least one portion/d), which ranged from 0·04 to 26·8 % is not surprising, as it corroborates with evidence that Western Africa is far from meeting their recommended five portions (400 g) of fruits and vegetables per d^(20,73)^. Similarly, another study also reported that adolescents in forty-nine low-income countries did not consume as many fruits and vegetables as recommended^(74)^. The inadequacy of vegetable consumption suggests that West African countries are at a higher risk of non-communicable diseases and increased prevalence of micronutrient malnutrition.

### Interventions that promote vegetable intake

Nutrition and health education interventions delivered in various forms particularly with visual aids were the dominant vegetable intake interventions given to the SAC and adolescents. Interventions that had additional hands-on/practical components like gardening and gamification were less prominent. This corroborates the report from a systematic review on school-based health and nutrition interventions addressing the double burden of malnutrition and educational outcomes of adolescents in low- and middle-income countries^(75)^. The study reported a higher prevalence of nutrition education alone compared with having nutrition education along with other hands-on/practical intervention components that will actually facilitate positive dietary outcomes.

Although traditional nutrition education methods reportedly show good promise in these studies, there are still concerns regarding the extent to which this method achieves impactful outcomes among the age group in question if not combined with other practical components/activities^(76)^. The authors suggest that the design of nutrition and health education for young people should incorporate hands-on practical components within the environment^(77)^. School feeding is the largest social safety net for young people, with increasing institutional and political commitment in West Africa targeted for young people, while home gardening on the other hand is being advocated as a sustainable approach to improve food security in low-income settings^(8)^. Thus, integrating additional components like practical gardening sessions will not only promote behavioural change but also increase availability and accessibility and offer some additional fresh FV for consumption in a sustainable way.

Virtually, all nutrition-focused game interventions in literature were directed at children and adolescents, as they are important stakeholders in the game industry^(25,78)^. Integrating nutrition and health education into this youth-dominated industry is an innovation that will likely garner the interest of consumers^(79)^. Several nutrition incentive-based behaviour change interventions in literature were structured as a reward for positive behaviours^(80,81)^. It will be interesting to see how much evidence evolves to support the translation of points in nutrition education games into values usable in real life to influence behaviour changes as reported in our studies.

### Barriers and facilitators of vegetable consumption

Family (parental intake) and home environment (accessibility and availability) were the dominant factors influencing vegetable consumption among SAC and adolescents. This corresponds with reports from a systematic review on determinants of FV consumption among children and adolescents^(82,83)^. Another study reported that parental participation, when combined with digital interventions, improved teenagers’ dietary and physical activity behaviours^(84)^. Evaluation of the factors that affect children and adolescents’ intake of fruits and vegetables in various parts of the world has shown that parental intake and home accessibility and availability were consistently positively associated with intake^(82,85)^. A study among Tehrani teenagers revealed that motivation was significantly influenced by verbal encouragement, supervision and instructions from parents, family, relatives and friends^(86)^.

### Limitations of the study

There are limitations to this systematic review. First, it is difficult to compare results since different research employed different methods to measure vegetable consumption (e.g. some used a 24 h dietary recall, while others used an FFQ). Selection of appropriate risk of bias tool was challenging as studies on vegetable consumption employed distinct research designs. Furthermore, the reference period and response categories varied even among articles that used the same technique. Second, data heterogeneity was noted, which made comparing research challenging. Furthermore, the consumption indicators used in the various papers differed (some studies gave means, others percentages and yet others frequencies). In cases where fruits and vegetables were lumped together, it was difficult to ascertain the consumption of vegetables and also in those studies that assessed vegetable consumption; most of them grouped the various types of vegetables together, while few studies disaggregated them. Finally, not all countries in the West African sub-region had published records of studies on vegetable consumption among these age groups. Hence our review is limited to the results of the studies from the countries where we found published studies.

### Conclusion

This review indicates an inadequate intake of vegetables among SAC and adolescents in countries located in West Africa. Inadequate vegetable intake may contribute to poor health outcomes, especially micronutrient inadequacies, and other nutritional problems associated with low intake of vegetables. Therefore, it is crucial to discover the most effective programmes that can early on influence children and adolescents’ healthy eating habits and in particular their vegetable intake. The interventions found in the articles reviewed include the use of nutrition education, gamification, school-based fruit stall and school gardens and complementary nutrition education. These interventions seemed to influence vegetable consumption and nutrition knowledge of the SAC and adolescents. More empirical multi-component and innovative studies to improve vegetable consumption as a food group are urgently needed in the West Africa sub-region. Such studies should include food system factors that will make vegetables more available, accessible and desirable for children and adolescents. For example, parental participation (related to home food environment), vegetable gardening (production at home and/or school), food demonstrations (cooking/recipe development) and school meals, all linked to interactive nutrition education lessons among others, are important factors to consider in such studies. Gamification of nutrition education as a means to promote better dietary habits among this age group seems to also be a promising strategy to explore as well.

It is also important that the types of vegetables studied should be disaggregated, to identify the types of vegetables (leafy and non-leafy) that are commonly and less commonly consumed among the target group. Studies that focus on quantification of different types of vegetables consumed are also needed. In countries where studies have been carried out, it is encouraged that they are published so that the progress made in regions/countries is updated.

## Supporting information

Igbokwe et al. supplementary material Igbokwe et al. supplementary material

## Supplementary material

For supplementary material/s referred to in this article, please visit https://doi.org/10.1017/S0007114524003301.

## References

[1] United Nations Systems Standing Committee on Nutrition (UNSCN) (2017) Schools as a System to Improve Nutrition A New Statement for School-Based Food and Nutrition Interventions. Discussion Paper, September, 2017. https://www.unscn.org/uploads/web/news/document/School-Paper-EN-WEB.pdf (accessed October 2024).

[2] Schwarzenberg SJ & Georgieff MK (2018) Advocacy for improving nutrition in the first 1000 days to support childhood development and adult health. Pediatrics 141, e20173716. 29358479 10.1542/peds.2017-3716

[3] Berti C & Agostoni C (2017) *Programming Long-Term Health: Establishing Healthy Eating Patterns in Early Infancy. Early Nutrition and Long-Term Health*. Cambridge: Woodhead Publishing. pp. 427–470. ISBN 9780081001684.

[4] Scaglioni S , De Cosmi V , Ciappolino V , et al. (2018) Factors influencing children’s eating behaviours. Nutrients 10, 706. 29857549 10.3390/nu10060706 PMC6024598

[5] Ishdorj A , Jensen H & Crepinsek M (2013) Children’s consumption of fruits and vegetable: do school environment and policies affect choice at school and away from school? Appl Econ Perspective Policy 35, 341–359.

[6] Ochola S & Masibo PK (2014) Dietary intake of school children and adolescents in developing countries. Ann Nutr Metab 64, 24–40. 25341871 10.1159/000365125

[7] Erkan TÃ (2011) Adolescent nutrition. Turk Pediatr Archive/Turk PediatriArsivi 46, 49–53.

[8] Iheme GO (2021) The under-nutrition situation of school age children in Nigeria; a systematic review. Curr Nutr Food Sci 17, 826–832. 10.2174/1573401317666210216114311

[9] World Food Programme (2022) State of School Feeding Worldwide 2022. Rome: World Food Programme.

[10] Yip C , Chan W & Fielding R (2019) The associations of fruit and vegetable intakes with burden of diseases: a systematic review of meta-analyses. J Acad Nutr Diet 119, 464–481. 30639206 10.1016/j.jand.2018.11.007

[11] Afshin A , Sur PJ , Fay KA , et al. (2019) Health effects of dietary risks in 195 countries, 1990–2017: a systematic analysis for the Global Burden of Disease Study 2017. Lancet 393, 1958–1972. 30954305 10.1016/S0140-6736(19)30041-8 PMC6899507

[12] Harika R , Faber M , Samuel F , et al. (2017) Are low intakes and deficiencies in iron, vitamin A, zinc, and iodine of public health concern in Ethiopian, Kenyan, Nigerian, and South African children and adolescents? Food Nutr Bull 38, 405–427. 28682645 10.1177/0379572117715818

[13] World Health Organization (2020) ‘Obesity and Overweight.’ WHO. https://www.who.int/news-room/fact-sheets/detail/obesity-and-overweight (accessed October, 2024).

[14] 2020 Global Nutrition Report: Action on equity to end malnutrition. Bristol, UK: Development Initiatives. https://media.globalnutritionreport.org/documents/2020_Global_Nutrition_Report_2hrssKo.pdf (accessed October, 2024).

[15] Lopes PH , Donne KA , George KK , et al. (2018) Fruit and vegetable, sugar-sweetened beverage consumption among kindergartners in Accra Metropolitan Area, Ghana: a cross-sectional study. Eur J Prev Med 6, 53–57.

[16] World Bank (2024) Western & Central Africa. World Bank. https://www.worldbank.org/en/region/afr/western-and-central-africa (accessed October, 2024).

[17] Hall J , Moore S , Harper S , et al. (2009) Global variability in fruit and vegetable consumption. Am J Med 36, 402–409. 10.1016/j.amepre.2009.01.029 19362694

[18] World Health Organization (2005) Fruit and Vegetables for Health: Report of a Joint FAO/WHO Workshop, 1–3 September 2004, Kobe, Japan (accessed March 2023).

[19] Spence AC , Karen JC , Sandrine L , et al. (2018) Early childhood vegetable, fruit, and discretionary food intakes do not meet dietary guidelines, but do show socioeconomic differences and tracking over time. J Acad Nutr Diet 118, 1634–1643. 29482964 10.1016/j.jand.2017.12.009

[20] Padget A & Briley ME (2005) Dietary intakes at child-care centers in Central Texas fail to meet food guide pyramid recommendations. J Am Dietetic Assoc 105, 790–793. 10.1016/j.jada.2005.02.002 15883557

[21] Han X , Ding S , Lu J , et al. (2022) Global, regional, and national burdens of common micronutrient deficiencies from 1990 to 2019: a secondary trend analysis based on the Global Burden of Disease 2019 study. E Clin Med 44, 101299. 10.1016/j.eclinm.2022.101299 PMC8850322 35198923

[22] Dijkxhoorn Y , De B , Piters S , et al. (2021) Enhancing Fruit and Vegetable Consumption in Low-and Middle Income Countries through a Food Systems Approach. https://edepot.wur.nl/555408 (accessed December, 2023).

[23] Micha R , Khatibzadeh S , Shi P , et al. (2015) Global Burden of Diseases Nutrition and Chronic Diseases Expert Group (NutriCoDE), Global, regional and national consumption of major food groups in 1990 and 2010: a systematic analysis including 266 country-specific nutrition surveys worldwide. BMJ Open 5, e008705. 10.1136/bmjopen-2015-008705 PMC4593162 26408285

[24] Miller V , Yusuf S , Chow CK , et al. (2016) Availability, affordability, and consumption of fruits and vegetables in 18 countries across income levels: findings from the Prospective Urban Rural Epidemiology (PURE) study. Lancet Global Health 4, e695–e703. 27567348 10.1016/S2214-109X(16)30186-3

[25] Hoelscher DM , Evans A , Parcel G , et al. (2002) Designing effective nutrition interventions for adolescents. J Am Dietetic Assoc 102, S52–S63. 10.1016/s0002-8223(02)90422-0 11902389

[26] Jacob CM , Hardy-Johnson PL , Inskip HM , et al. (2021) A systematic review and meta-analysis of school-based interventions with health education to reduce body mass index in adolescents aged 10 to 19 years. Int J Behav Nutr Phys Act 18, 1–22. 10.1186/S12966-020-01065-9/FIGURES/3 33397403 PMC7784329

[27] Saavedra JM & Prentice AM (2022) Nutrition in school-age children: a rationale for revisiting priorities. Nutr Rev 81, 823–843. 10.1093/nutrit/nuac089 PMC10251301 36346900

[28] Moola S , Munn Z , Tufanaru C , et al. (2017) Chapter 7: Systematic reviews of etiology and risk. In *Joanna Briggs Institute Reviewer’s Manual* [ E Aromataris and Z Munn , editors]. Adelaide, Australia: The Joanna Briggs Institute.

[29] Ilo JG , Onabanjo OO , Badejo CO , et al. (2022) The dietary pattern and hemoglobin status of school-age children in Odeda local government area of Ogun State in Nigeria. Int J Food Agric Nat Resour 03, 8–13.

[30] Anyiam PN , Nwuke CP , Adimuko GC , et al. (2022) Dietary intake and nutritional status of school-children in Umudike, South-East Nigeria during Covid-19 Context. Int J Nutr Sci 7, 81–89.

[31] Amu EO , Olatona FA & Deji SA (2017) A comparative study of food consumption pattern among public and private primary school children in Ojodu Local Government Area, Lagos State, Nigeria. Njfp 8, 65–70.

[32] Fadeiye EO & Adekanmbi ET (2020) Fruit and vegetable consumption among primary school pupils of Egbeda Local Government Area, Oyo State, Nigeria. Ife J Agricult 9, 103–115.

[33] Adeomi AA , Aliyu SM & Sabageh AO (2020) Eating pattern, dietary diversity and nutritional status of children and adolescents residing in Orphanages in Southwestern Nigeria. J Community Med Primary Health Care 32, 59–69.

[34] Agugo UA , Asinobi CO & Afam-Anene O (2019) Impact of food consumption pattern on the Body Mass Index (BMI) of school children (5–12 years) in selected motherless and orphanage homes in Imo State. J Nutr Sci Res 4, 135.

[35] John-Akinola YO , Akano OO & Akinwale O (2021) Supporting a participatory process for evidence on healthy eating to promote healthy diet among children: an illustration from Nigeria. Health Behav Policy Rev 8, 269–276.

[36] Adeniyi OF , Fagbenro GT & Olatona FA (2019) Overweight and obesity among school-aged children and maternal preventive practices against childhood obesity in select local government areas of Lagos, Southwest, Nigeria. Int J MCH AIDS 8, 70–79. 31321148 10.21106/ijma.273 PMC6630490

[37] Akinola IJ , Odugbemi B , Bakare OQ , et al. (2022) Dietary habits, physical activity and sleep pattern among in-school adolescents in Lagos, Nigeria. Ann Health Res 8, 63–73.

[38] Olumakaiye MF (2013) Dietary diversity as a correlate of undernutrition among school-age children in southwestern Nigeria. J Child Nutr Manag 1, 1–32.

[39] Ayogu R (2019) Energy and nutrient intakes of rural Nigerian schoolchildren: relationship with dietary diversity. Food Nutr Bull 40, 241–253. 31064219 10.1177/0379572119833854

[40] Ibeanu VN , Edeh GC & Ani PN (2020) Evidence-based strategy for prevention of hidden hunger among adolescents in a suburb of Nigeria. BMC Public Health 20, 1–10. 33172420 10.1186/s12889-020-09729-8 PMC7654145

[41] Menakaya NC & Menakaya IN (2022) Qualitative study exploring perceptions, attitudes and practices of adolescent university students in Lagos, Nigeria, towards a healthy lifestyle. Afr J Primary Health Care Fam Med 14, 1–12. 10.4102/phcfm.v14i1.3577 PMC9634713 36331197

[42] Silva OO , Olayinka OA & Tinuola OO (2017) Knowledge and consumption of fruits and vegetables among secondary school students of Obele Community Junior High School, Surulere, Lagos State, Nigeria. J Clin Sci 14, 68–73.

[43] Olatona FA , Ogide PI , Abikoye ET , et al. (2020) Dietary Patterns, Nutritional Knowledge and Status of Adolescents in Lagos, Nigeria. Research Square. 10.21203/rs.3.rs-18023/v1 (accessed January 2023). PMC10521850 37767409

[44] Anaemene D & Ogunkunle M (2020) Overweight status and dietary habit of children attending private schools in Ado Odo Ota, South Western Nigeria. Afr J Food Agric Nutr Dev 20, 22.

[45] Shapu CR , Ismail S , Poh-Ying L , et al. (2022) Impact of health education intervention on dietary practice among adolescent girls in government secondary schools Maiduguri: a cluster randomized control trial: a cluster randomized control trial on health education intervention on dietary practice. Niger Health J 22, 417–427.

[46] Ogunkunle MO & Oludele AS (2013) Food intake and meal pattern of adolescents in school in Ila-Orangun, South-West Nigeria. S Afr J Clin Nutr 26, 188–193.

[47] Wordu GO & Wachukwu-Chikodi HI (2019) Dietary intake and prevalence of adolescent hypertensive in Port Harcourt, Nigeria. Int J Res Granthaalayah 7, 22–29.

[48] Uba DS , Islam MR , Haque MI , et al. (2020) Nutritional status of adolescent girls in a selected secondary school of north-eastern part of Nigeria. Middle East J Rehabil Health Stud. 7(4):e104331. 10.5812/mejrh.104331.

[49] Wordu GO & Orisa CA (2021) Diet, physical activity and food consumption pattern of adolescent girls in Port Harcourt, Rivers State, Nigeria. Eur J Nutr Food Saf 13, 38–47.

[50] Nnebue CC , Ilika AL , Uwakwe KA , et al. (2016) Feeding practices and determinant of the nutritional status of pupils in a public primary school in Aladinma Owerri, Nigeria. Int J Clin Nutr 4, 12–18.

[51] Sanusi RA , Yusuf FK & Ejoh SI (2015) Assessment of dietary diversity of in-school adolescents in Ibadan, Oyo State, Nigeria. West Afr J Food Nutr 12, 69–77.

[52] Ezezika O , Oh J , Edeagu N , et al. (2018) Gamification of nutrition: a preliminary study on the impact of gamification on nutrition knowledge, attitude, and behaviour of adolescents in Nigeria. Nutr Health 24, 137–144. 29974803 10.1177/0260106018782211

[53] Seidu AA , Aboagye RG , Frimpong JB , et al. (2021) Determinants of fruits and vegetables consumption among in-school adolescents in Ghana. Adolescents 1, 199–211.

[54] Yaméogo T , Sombié I , Kyelem C , et al. (2018) Determinants of fruit and vegetables intake among secondary school pupils in the city of Bobo-Dioulasso (Burkina Faso): a cross-sectional study. Open J Intern Med 8, 1–9.

[55] Giguère-Johnson M , Ward S & Ndéné Ndiaye A (2021) Dietary intake and food behaviours of Senegalese adolescent girls. BMC Nutr 7, 41. 34289906 10.1186/s40795-021-00436-0 PMC8296647

[56] Nago ES , Lachat CK & Huybregts L (2010) Food, energy, and macronutrient contribution of out-of-home foods in school-going adolescents in Cotonou, Benin. Br J Nutr 103, 281–288. 19818195 10.1017/S0007114509991668

[57] Owusu A , Murdock PO & Weatherby NL (2007) Measuring nutritional intake of adolescents in Ghana, West Africa. Int Electron J Health Educ 10, 104–113.

[58] Sagbo H , Kpodji P , Bakai TA , et al. (2022) Socio-economic determinants of healthy behaviours among primary schoolchildren and adolescents in Lokossa district of southern Benin. Int Health 15, 265–273. 10.1093/inthealth/ihac018 PMC10153559 35488369

[59] Doku D , Koivusilta L & Raisamo S (2013) Socio-economic differences in adolescents’ breakfast eating, fruit and vegetable consumption, and physical activity in Ghana. Public Health Nutr 16, 864–872. 22030213 10.1017/S136898001100276X PMC10271242

[60] Abizari AR , Azupogo F , Nagasu M , et al. (2017) Seasonality affects dietary diversity of school-age children in northern Ghana. PLOS ONE 12, e0183206. 28806418 10.1371/journal.pone.0183206 PMC5555613

[61] Hormenu T (2022) Dietary intake and its associated factors among in-school adolescents in Ghana. PLoS ONE 17, e0268319. 35552563 10.1371/journal.pone.0268319 PMC9097987

[62] Dabone C , Delisle H & Receveur O (2013) Predisposing, facilitating and reinforcing factors of healthy and unhealthy food consumption in schoolchildren: a study in Ouagadougou, Burkina Faso. Global Health Promot 20, 68–77. 10.1177/1757975913476905 23563781

[63] Fiorentino M , Landais E , Bastard G , et al. (2016) Nutrient intake is insufficient among Senegalese Urban School children and adolescents: results from two 24 h recalls in state primary schools in Dakar. Nutrients 8, 650. 27775598 10.3390/nu8100650 PMC5084037

[64] Alangea OD , Aryeetey RN & Gray HL (2018) Dietary patterns and associated risk factors among school-age children in urban Ghana. BMC Nutr 4, 22. 32153885 10.1186/s40795-018-0230-2 PMC7050789

[65] Nago E & Chabi SM (2019) A pilot school-based intervention to increase fruit intake in adolescents in Urban Benin. Food Public Health 9, 111–118.

[66] Otuneye AT , Ahmed PA , Abdulkarim AA , et al. (2017) Relationship between dietary habits and nutritional status among adolescents in Abuja municipal area council of Nigeria. Niger J Paediatrics 44, 128–135

[67] Uzosike TCJ , Okeafor I & Mezie-Okoye M (2020) Dietary diversity, nutritional status and academic performance of pupils in public primary schools in Port Harcourt Metropolis. J Community Med Primary Healthcare 32, 42–56.

[68] Schreinemachers MS , Ouedraogo S , Diagbouga A , et al. (2019) Impact of school gardens and complementary nutrition education in Burkina Faso. J Dev Eff 11, 132–145.

[69] FAO (2018) Dietary Assessment: A Resource Guide to Method Selection and Application in Low Resource Settings. Rome: FAO.

[70] Micha R , Coates J , Leclercq C , et al. (2018) Global dietary surveillance: data gaps and challenges. Food Nutr Bull 39, 175–205. 10.1177/0379572117752986. 29478333

[71] Gurugubelli VS , Fang H , Shikany JM , et al. (2022) A review of harmonization methods for studying dietary patterns. Smart Health 23, 100263. 10.1016/j.smhl.2021.100263. 35252528 PMC8896407

[72] Cui Q , Xia Y , Wu Q , et al. (2021) A meta-analysis of the reproducibility of food frequency questionnaires in nutritional epidemiological studies. Int J Behav Nutr Physical Activity 18, 1–18. 10.1186/s12966-020-01078-4 PMC7802360 33430897

[73] World Health Organization (2020) Healthy Diet. https://www.who.int/news-room/fact-sheets/detail/healthy-diet

[74] Darfour-Oduro SA , Buchner DM , Andrade JE , et al. (2018) A comparative study of fruits and vegetables consumption and physical activity among adolescents in 49 Low-and-Middle-Income Countries. Sci Rep 8, 1623. 29374197 10.1038/s41598-018-19956-0 PMC5785955

[75] Shinde S , Wang D , Moulton GE , et al. (2023) School-based health and nutrition interventions addressing double burden of malnutrition and educational outcomes of adolescents in low- and middle-income countries: a systematic review. Matern Child Nutr 10.1111/mcn.1343 PMC12208896 36994620

[76] Miguel-Berges ML , Santaliestra-Pasias AM , Mouratidou T , et al. (2017) Associations between food and beverage consumption and different types of sedentary behaviours in European preschoolers: the ToyBox-study. Eur J Nutr 56, 1939–1951. 10.1007/s00394-016-1236-7 27312566

[77] Azevedo J , Padrão P , Gregório MJ , et al. (2019) A web-based gamification program to improve nutrition literacy in families of 3- to 5-year-old children: the Nutriscience project. J Nutr Educ Behav 51, 326–334. 10.1016/j.jneb.2018.10.008 30579894

[78] Rosati R , Regini L , Pauls A , et al. (2024) Gamification in nutrition education: the impact and the acceptance of digital game-based intervention for improving nutritional habits. J Comput Educ 10.1007/s40692-024-00314-1

[79] Edwards EA , Lumsden J , Rivas C , et al. (2016) Gamification for health promotion: systematic review of behaviour change techniques in smartphone apps. BMJ Open 6, e012447. 10.1136/bmjopen-2016-012447 PMC5073629 27707829

[80] Spring B , Schneider K , Mcfadden HG , et al. (2012) Multiple behavior changes in diet and activity: a randomized controlled trial using mobile technology. Arch Intern Med 172, 789–796. 10.1001/archinternmed.2012.1044 22636824 PMC3402206

[81] Mantzari E , Vogt F , Shemilt I , et al. (2015) Personal financial incentives for changing habitual health-related behaviors: a systematic review and meta-analysis. Prev Med 75, 75–85. 10.1016/j.ypmed.2015.03.001 25843244 PMC4728181

[82] Mette R , Rikke K , Knut-Inge K , et al. (2006) Determinants of fruit and vegetable consumption among children and adolescents: a review of the literature. Part I: quantitative studies. Int J Behav Nutr Phys Act 3, 22. 16904006 10.1186/1479-5868-3-22 PMC1564033

[83] Fadeiye EO , Popoola BR , Emuoke DK , et al. (2019) Factors influencing fruit consumption among undergraduates in Obafemi Awolowo University, Ile-Ife, Osun state, Nigeria. Ife J Agric 31, 80–89.

[84] Rose T , Mary B , Chandni MJ , et al. (2017) A systematic review of digital interventions for improving the diet and physical activity behaviors of adolescents. J Adolesc Health 61, 669–677. 28822682 10.1016/j.jadohealth.2017.05.024 PMC5702542

[85] Sumonja S & Novakovic B (2012) Determinants of fruit, vegetable, and dairy consumption in a sample of school children, Northern Serbia. Prev Chronic Dis 31, E178. 10.5888/pcd10.130072 PMC3816602 24176082

[86] Rakhshanderou S , Ramezankhani A , Mehrabi Y , et al. (2014) Determinants of fruit and vegetable consumption among Tehranian adolescents: a qualitative research. J Res Med Sci 19, 482–489. 25197287 PMC4155700

