# Supplementary material for: Vegetable consumption and promotion among school-age children and adolescents in West Africa: a systematic review and narrative synthesis
Source: Br J Nutr. 2025 Jan 10;133(3):408–21. doi: 10.1017/S0007114524003301 (PMC11946038; doi:10.1017/S0007114524003301)
Supplement: Igbokwe et al. supplementary material [file S0007114524003301sup001.docx]

**SUPPLEMENTARY FILE 1**

**Quality assessment of the reviews using JBI Critical Appraisal Checklist**

| **Included Reviews** | **Questions** | | | | | | | | **Number of Criteria met** |
| --- | --- | --- | --- | --- | --- | --- | --- | --- | --- |
|  | 1 | 2 | 3 | 4 | 5 | 6 | 7 | 8 |  |
| Ilo*et al.,* 2022 | N | N | Y | Y | Y | NA | NA | 1 | 4 |
| Anyiam*et al.,* 2022 | N | Y | Y | Y | NA | NA | NA | Y | 4 |
| Amu *et al*., 2017 | Y | Y | Y | Y | NA | NA | NA | Y | 5 |
| Fadeiye and Adekanmbi, 2020 | Y | Y | Y | Y | NA | NA | NA | Y | 5 |
| Adeomi*et al*., 2020 | Y | Y | Y | Y | NA | NA | NA | Y | 5 |
| Agugo*et al*., 2019 | Y | N | Y | Y | NA | NA | NA | Y | 4 |
| John-Akinola *et al*., 2021 | Y | Y | Y | Y | NA | NA | NA | Y | 5 |
| Adeniyi *et al*., 2019 | Y | Y | Y | N | NA | NA | NA | Y | 4 |
| Akinola *et al*., 2022 | Y | Y | Y | Y | NA | NA | NA | Y | 5 |
| Olumakaiye, 2013 | Y | N | Y | Y | NA | NA | NA | Y | 4 |
| Ayogu, 2019 | Y | Y | Y | Y | NA | NA | NA | Y | 5 |
| Ibeanu*et al*., 2020 | Y | Y | Y | Y | N | Y | Y | Y | 7 |
| Menakaya and Menakaya, 2022 | N | Y | Y | U | Y | NA | NA | Y | 4 |
| Silva *et al*., 2017 | Y | Y | Y | N | NA | NA | NA | N | 3 |
| Olatona*et al*., 2020 | Y | Y | Y | Y | NA | NA | NA | Y | 5 |
| Anaemene and Ogunkunle, 2020 | Y | Y | Y | Y | Y | NA | NA | Y | 6 |
| Shapu*et al*., 2022 | Y | N | Y | Y | N | Y | Y | Y | 6 |
| Ogunkunle and Oludele, 2013 | Y | Y | Y | Y | NA | NA | NA | Y | 5 |
| Wordu and Wachukwu- Chikodi, 2019 | Y | N | Y | N | NA | NA | NA | Y | 3 |
| Uba*et al*., 2020 | Y | Y | Y | Y | NA | NA | NA | Y | 5 |
| Wordu and Orisa, 2021 | Y | N | Y | Y | NA | NA | NA | Y | 4 |
| Nnebue*et al*., 2016 | Y | Y | Y | N | NA | NA | NA | Y | 4 |

**Quality assessment of the reviews using JBICritical Appraisal Checklist (cont’d)**

| **Included Reviews** | **Questions** | | | | | | | | **Number of Criteria met** |
| --- | --- | --- | --- | --- | --- | --- | --- | --- | --- |
|  | 1 | 2 | 3 | 4 | 5 | 6 | 7 | 8 |  |
| Sanusi *et al*., 2015 | Y | Y | Y | Y | NA | NA | NA | Y | 5 |
| Ezezika*et al*., 2018 | N | Y | Y | Y | NA | NA | NA | Y | 4 |
| Seidu*et al*., 2021 | Y | Y | Y | Y | NA | NA | NA | Y | 5 |
| Yaméogo*et al*., 2018 | Y | N | Y | Y | NA | NA | NA | Y | 4 |
| Giguère-Johnson *et al*., 2021 | N | Y | Y | Y | NA | NA | NA | Y | 4 |
| Nago*et al*., 2009 | Y | Y | Y | Y | NA | NA | NA | Y | 5 |
| Owusu *et al*., 2007 | Y | Y | Y | Y | NA | NA | NA | N | 4 |
| Sagbo*et al*., 2022 | Y | N | Y | Y | NA | NA | NA | Y | 4 |
| Doku*et al*., 2011 | Y | Y | Y | Y | NA | NA | NA | Y | 5 |
| Abizari*et al*., 2017 | N | Y | Y | Y | N | Y | Y | Y | 6 |
| Hormenu 2022 | Y | Y | Y | Y | NA | NA | NA | Y | 5 |
| Dabone*et al*., 2013 | N | Y | Y | Y | NA | NA | NA | Y | 4 |
| Fiorentino*et al*., 2016 | Y | Y | Y | Y | NA | NA | NA | Y | 5 |
| Alangea*et al*., 2018 | Y | Y | Y | Y | NA | NA | NA | Y | 5 |
| Nago and Chabi, 2019 | N | Y | Y | Y | N | N | N | Y | 4 |
| Otuneye*et al*., 2017 | Y | Y | Y | Y | Y | NA | NA | Y | 6 |
| Uzosike*et al*., 2020 | Y | Y | Y | Y | N | NA | NA | Y | 5 |
| Schreinemachers*et al*., 2019 | Y | Y | Y | Y | Y | Y | N | Y | 7 |

JBI Key: 1 = Yes

2 = No

3 = Unsure

1. = Not applicable.

Areas assessed are numbered 1 to 8 on horizontal axis; 1-described as randomized, 2-clear definition of study inclusion criteria, 3-description of study subjects and settings, 4-outcomes/exposures measured in a valid and reliable way, 5-identification of confounding factors and strategies, 6-sufficient description for comparison groups, 7-follow-up done for a sufficient time period, 8- appropriate statistical analysis.
